# RGD-PEG-PLA Delivers MiR-133 to Infarct Lesions of Acute Myocardial Infarction Model Rats for Cardiac Protection

**DOI:** 10.3390/pharmaceutics12060575

**Published:** 2020-06-21

**Authors:** Bixi Sun, Shuwen Liu, Rubin Hao, Xinyue Dong, Lanbo Fu, Bing Han

**Affiliations:** Department of Biopharmacy, School of Pharmaceutical Sciences, Jilin University, Changchun 130021, China; sunbq19@mails.jlu.edu.cn (B.S.); liushuwenjlu@126.com (S.L.); rubinhao@163.com (R.H.); dong_xinyue@126.com (X.D.); fu_lanbo@163.com (L.F.)

**Keywords:** acute myocardial infarction, miRNA-133, nanoparticles, arginine-glycine-aspartic acid tripeptide (RGD), targeting

## Abstract

Studies have shown that microRNA-133 (miR-133) plays a positive role in the growth of cardiac myocytes, the maintenance of cardiac homeostasis, and the recovery of cardiac function, which is of great significance for the recovery of acute myocardial infarction. However, the delivery of miRNA to the site of action remains a challenge at present. The purpose of this study was to design an ideal carrier to facilitate the delivery of miR-133 to the infarct lesion for cardiac protection. A disease model was constructed by ligating the left anterior descending coronary artery of rats, and polyethylene glycol (PEG)-polylactic acid (PLA) nanoparticles modified with arginine-glycine-aspartic acid tripeptide (RGD) carrying miR-133 were injected via the tail vein. The effects of miR-133 were evaluated from multiple perspectives, including cardiac function, blood indexes, histopathology, and myocardial cell apoptosis. The results showed that RGD-PEG-PLA maintained a high level of distribution in the hearts of model rats, indicating the role of the carrier in targeting the heart infarction lesions. RGD-PEG-PLA/miR-133 alleviated cardiac histopathological changes, reduced the apoptosis of cardiomyocytes, and reduced the levels of factors associated with myocardial injury. Studies on the mechanism of miR-133 by immunohistochemistry and polymerase chain reaction demonstrated that the expression level of Sirtuin3 (SIRT3) was increased and that the expression of adenosine monophosphate activated protein kinase (AMPK) decreased in myocardial tissue. In summary, the delivery of miR-133 by RGD-PEG-PLA carrier can achieve cardiac lesion accumulation, thereby improving the cardiac function damage and reducing the myocardial infarction area. The inhibition of cardiomyocyte apoptosis, inflammation, and oxidative stress plays a protective role in the heart. The mechanism may be related to the regulation of the SIRT3/AMPK pathway.

## 1. Introduction

The high morbidity and mortality of cardiovascular diseases pose a serious threat to the health of elderly people [1], and impose a heavy medical burden on society. With the intensification of the aging process worldwide, acute myocardial infarction (AMI) has gradually become the main cause of death [2]. AMI is mainly manifested as myocardial necrosis and impairment of cardiac function caused by irreversible ventricular remodeling [3], which is ischemic heart disease caused by local vascular blockage of the heart [4]. AMI triggers the hypoxic microenvironment and causes extensive myocardial injury. Since myocardial cells are terminally differentiated cells with no or almost no regenerative potential, preventing the loss of myocardial cells after AMI injury is a critical clinical treatment strategy. Although the current treatment methods, such as surgical intervention and thrombolysis, can temporarily relieve the myocardial ischemia and hypoxia state, they cannot repair the damaged myocardial infarction area and may cause ischemia, tissue damage, and other irreversible myocardial ischemia-reperfusion injuries (MIRI); furthermore, the therapeutic effect is often unsatisfactory [5].

Researchers have gradually turned their attention to another novel treatment, that is, repairing myocardial cells in the dying state of the marginal area after infarction with special noncoding RNA and proteins to reduce the scope of infarction [6]. MicroRNAs (miRNAs) play an important regulatory role in regulating the microenvironment of myocardial tissue and are widely involved in angiogenesis, apoptosis, and fibrosis after myocardial infarction, and miRNAs are expected to become biomarkers and potential therapeutic targets for AMI [7,8]. Among them, microRNA-133 (miR-133) inhibits the apoptosis of myocardial cells, reduces the scope of myocardial infarction, and exhibits a protective effect on the heart, which has been proven by an increasing number of researchers. Many researchers have demonstrated a positive role of miR-133 in the cardiovascular field in vivo and in vitro [3,9,10,11,12]. Song et al. confirmed that tanshinone IIA could relieve myocardial cell apoptosis by upregulating miR-133 [13]. He et al. demonstrated that when MIRI occurred, promoting the expression of miR-133a could inhibit cardiomyocyte apoptosis [14]. Bostjancic et al. concluded that compared with those of healthy adults, the most significant changes in the hearts of patients with myocardial infarction included the downregulation of miR-133a [15]. Xu et al. conducted that miR-133 had a protective effect on oxidative stress-induced myocardial apoptosis by downregulating caspase-9 and caspase-3 [16]. Other researchers directly delivered miR-133 into animal models of AMI and achieved better therapeutic effects. At present, some problems still need to be solved to fully elucidate the role of miR-133 in alleviating myocardial infarction [17]. First, miRNA is sensitive and is easily degraded by the enzyme system in the blood after intravenous or systemic administration. Second, miRNA affects multiple molecular pathways, and it is easy to act on other target organ tissues while treating the target organ, resulting in adverse effects. The delivery of miRNAs has been enhanced by nanoparticles to improve the therapeutic effect of cancer [18,19], but there are few studies on the effective delivery carrier of miRNAs in the heart field. Our study attempted to design a delivery carrier to aggregate miRNAs at the action site to enhance therapeutic activity.

In the treatment of cardiovascular diseases, the nanomaterials have shown attractive charm. Nanoparticles act as delivery systems, making it possible to provide treatments with drugs that are difficult to stabilize in body fluids, have poor solubility, or have short half-lives. Nanoparticles which are made of nontoxic, biodegradable polymers show promise in drug delivery use, which render multiple advantages, including: sustained-release, controlled-release, long-acting, targeting, higher-loading, and high-compliance drugs [20]. The enhanced permeability and retention (EPR) effect of nanoparticles at the myocardial infarction site is similar to that of tumor tissue [3,21], and accumulation at the infarction site can enable effective passive targeted therapy. Studies have shown that arginine-glycine-aspartic acid tripeptide (RGD) sequences can bind to activated platelets at the thrombus site but have no effect on circulating platelets, indicating good thrombus targeting [7,22]. Therefore, in this study, RGD-modified polyethylene glycol (PEG)-polylactic acid (PLA) was used as the carrier to deliver miR-133 to the heart lesion site, and miR-133 was delivered to the heart to exhibit protective effects on the cardiac muscle using a combination of the active and passive targeting strategies. This combination of targeting strategies is innovative to some extent and has not been reported by predecessors. The results confirmed that RGD-modified PEG-PLA showed a higher distribution in the heart and that RGD-PEG-PLA/miR-133 alleviated myocardial tissue injury and reduced the apoptosis of myocardial cells to a certain extent. By inhibiting myocardial cell apoptosis, inflammation, and oxidative stress, RGD-PEG-PLA/miR-133 plays a cardioprotective role. The mechanism may be related to the regulation of the Sirtuin3 (SIRT3)/ adenosine monophosphate activated protein kinase (AMPK) pathway. It has been proven that the RGD-PEG-PLA carrier makes miR-133 exhibit more protective effects on cardiac infarction, thus providing a new carrier option for the application of miRNA in the field of AMI.

## 2. Materials and Methods

### 2.1. Materials

A Terminal-deoxynucleoitidyl Transferase-Mediated Nick End Labeling (TUNEL) in situ detection kit was obtained from Abcam (Cambridge, MA, USA). A bicinchoninic acid (BCA) protein determination kit and sodium dodecyl sulphate-polyacrylamide gel (SDS-PAGE) kit were purchased from Solarbio Science & Technology Co., LTD. (Beijing, China). SIRT3 antibody was obtained from Proteintech (Chicago, IL, USA). AMPK antibody was obtained from Bioworld (Dublin, OH, USA). Pentobarbital sodium and Horseradish peroxidase (HRP)-labeled goat anti-rabbit immunoglobulin G (IgG) were obtained from Zs-Bio Co., LTD. (Beijing, China). Ristocetin-induced platelet agglutination (RIPA) lysate was obtained from Beyotime Biotechnology Co., LTD. (Shanghai, China). A Quant-iT™ RiboGreen kit was obtained from Invitrogen Biotechnology Co., LTD. (Shanghai, China). NHS-PEG3400-PLA2000 polymer was obtained from Xinqiao Biotechnology Co., LTD. (Zhejiang, China). MPEG2000-PLA2000 polymer was obtained from Daigang Biotechnology Co., LTD. (Shandong, China). Moreover, all other chemicals used in this study were of molecular biology grades and commercially available.

### 2.2. Animals

Ninety male Wistar rats with body masses of 220–260 g were supplied by the animal experiment center of the College of Pharmacy, Jilin University. The rats were provided with food and water under standard feeding conditions, including suitable humidity, good ventilation, and 12 h light and dark cycles. The Animal Ethics Committee of Jilin University approved all animal experiments described in this study (code: 20180024, March 2018).

### 2.3. Preparation and Characterization of Nanoparticles

The synthesis of RGD-PEG-PLA conjugate was based on the methods by other researchers [23,24]. N-hydroxysuccinimide-PEG-PLA (540 mg) and RGD (35 mg) were reacted in anhydrous N,N-Dimethyl formamide (DMF, containing the amount of triethylamine equivalent to the RGD), and the system was stirred at room temperature for 24 h. The reaction mixture was dialyzed against deionized water for 24 h to remove unbound RGD (MWCO 3500 Da), the conjugation efficiency was 40.3%. The solution was immediately lyophilized for further study. The synthesis of miR-133-loaded RGD-PEG-PLA nanoparticles was based on the method we previously reported and was prepared by the *w*/*o*/*w* method [25]. In brief, miR-133 and spermidine with an N/P ratio of 10:1 were mixed in DNase/RNase-free water and kept at room temperature for 15 min to form a spermidine/miR-133 complex. RGD-PEG-PLA (25 mg) and mPEG-PLA (1:9, *w*:*w*) were dissolved in 2 mL of dichloromethane (DCM), and then, the spermine/miR-133 complex was added dropwise to the DCM mixture. The mixture was placed in an ice solution using a water bath and sonicated for 60 s to emulsify it. Then, 20 mL of 2.5% strength polyvinyl alcohol (*w*:*v*) aqueous solution was added and stirred, and the organic solvent was removed by rotary evaporation. The nanoparticles were centrifuged at 21,000× *g* for 45 min and then washed three times with ultrapure water. 1,1-dioctadecyl-3,3,3,3-tetramethylindotricarbocyaine iodide (DIR) was added to the oil phase to generate DIR-labeled nanoparticles to prepare blank nanoparticles. The particle size distribution and surface zeta potential of PEG-PLA/miRNA and RGD-PEG-PLA/miRNA were measured by dynamic light scattering (Nano ZS-3000, Malvern, UK) in the distilled water. Nanoparticles were imaged by transmission electron microscopy (TEM H-7650, Hitachi, Japan). The encapsulation efficiency of miRNA loading was tested with a Quant-iT™ RiboGreen kit.

### 2.4. Establishment of AMI Model in Rats and Administration

The AMI model was established by the permanent ligation of left coronary artery method [3,10,26]. The rats were anesthetized and fixed, and the cannula was inserted into the airway obliquely upward. If the gauze was blown with exhalation at the mouth of the cannula, the airway was successfully established. Then, the intubation was fixed and connected to an animal ventilator (HX-300S, Techman, Shanghai, China). The chest cavity was opened, the thoracotomy was placed between the third and fourth ribs of the rat, and the left anterior descending coronary artery (LAD) was ligated. With the ligation site and the subsequent myocardium turning white or blue as the standard, modeling was considered successful if the ST-segment of the electrocardiogram (ECG) continued to rise for more than half an hour. After the operation, the heart was reset, and intraperitoneal furosemide was injected. The spontaneous breathing state of the rats recovered and the intubation was pulled out.

The rats were randomly divided into the following 6 groups: the sham group, the model group, the positive drug group (aspirin, 10.4 mg/kg based on the treatment dose in myocardial infarction determined by previous researchers [27]), the miR-133 group, the PEG-PLA/miR-133 group, and the RGD-PEG-PLA/miR-133 group (2 mg/kg based on the treatment dose of miRNAs in myocardial infarction determined by previous researchers [28]), with 6 rats being intravenously injected in each group. The model group was prepared by the above modeling operation. All the operations of the sham group were the same as those of the model rats except that the thread was passed through the left coronary artery and knotted loosely. The sham group and the model group were given equal volumes of saline.

### 2.5. In Vivo Biodistribution Evaluation

Rats were intravenously injected with RGD-PEG-PLGA and PEG-PLGA nanoparticles loaded with the near-infrared dye DIR. At 0.5, 4, 12, and 36 h after injection, the rats were anesthetized and euthanized (*n* = 6 per group), and the spleen, liver, kidneys, heart, lungs, small intestine, large intestine, stomach, brain, fat tissue, and muscle were dissected. All samples were frozen at −80 °C until further processing. Using the previously reported method [29], in short, the tissues were thawed on ice and sliced, and 10% *w/v* deionized water was added to each sample. All the tissues (except the brain) were homogenized at maximum speed for 10 s in a magnetic bead homogenizer. 50 μL tissue homogenates were subsequently added to a 96-well plate containing 10 μL dimethylsulfoxide. The fluorescence intensity was read on a plate spectrophotometer (Infinite 2000, Tecan, Männedorf, Swiss) at 750 nm excitation/780 nm emission wavelength. The control group was constructed by adding a known quantity of DIR and vehicle to the tissue homogenate, and the calibration curves of each tissue type were plotted. All samples were in triplicate and the units were converted to ng DIR/g organization according to each control curve.

### 2.6. Assessment of Heart Functions

#### 2.6.1. Electrocardiogram

An electrocardiograph (BL-420S, Techman, Shanghai, China) of the rats was detected on the seventh day after AMI. The pathological Q wave numbers of leads I, aVL, and V1-V6v in rats were observed and recorded.

#### 2.6.2. Echocardiography

Rats were anesthetized for echocardiography detection on the seventh day after AMI, using a small animal ultrasound instrument (Vevo770, Visual Sonics, Toronto, ON, Canada). After the heart rate stabilized, a high-frequency probe (9L-D, 10.0 MHz) was placed on the front of the left chest for positioning. A short-axis parasternal image was obtained at the level of the mitral valve tendon. M-mode echocardiography was used to detect the left ventricular end-diastolic diameter (LVEDD), left ventricular end-systolic diameter (LVESD), left ventricular posterior wall thickness at end-diastole (LVPWD), left ventricular posterior wall thickness at end-systole (LVPWS), left ventricular ejection fraction (LVEF), and left ventricular fraction shortening (LVFS). All the original data were selected from three average values of consecutive cardiac cycle measurements.

### 2.7. Biochemical Indicators Detection

#### 2.7.1. Expression of Cardiac-Specific Markers

Seven days after AMI, the rats were anesthetized and blood was drawn from the abdominal aorta. A portion of the samples were centrifuged with a high-speed refrigerated centrifuge (5810R, Eppendorf, Hamburg, Germany) at 4000 r/min for 10 min, and the serum was divided into parts and stored at −80 °C (Haier, Shandong, China) for later storage. According to the instructions of the electrochemiluminescence kit, the expression of lactic dehydrogenase (LDH) (Jiancheng Bioengineering Co., LTD., Nanjing, China), creatine kinase isoenzyme (CK-MB) (Jingkang Biological Engineering Co., LTD., Shanghai, China), and cardiac troponin T (cTnT) (Jingkang Biological Engineering Co., LTD., Shanghai, China) were measured.

#### 2.7.2. Assessment of Oxidative Stress Markers

The activity of superoxide dismutase (SOD) in the reserve serum was determined by the xanthine oxidase method, the content of glutathione peroxidase (GSH-Px) was determined by the colorimetry method, and the content of malondialdehyde (MDA) was determined by the thiobarbital acid method. The three kits were purchased from Jingkang Biological Engineering Co., LTD. (Shanghai, China).

#### 2.7.3. Assessment of Inflammatory Cytokines

The remaining samples were centrifuged at 3000 r/min for 15 min, the serum was taken, and stored in a refrigerator at −80 °C. Enzyme-linked immunosorbent assay (ELISA) was used to detect the contents of tumor necrosis factor-α (TNF-α) (Jingkang Biological Engineering Co., LTD., Shanghai, China), interleukin-6 (IL-6) (Jiancheng Bioengineering Co., LTD., Nanjing, China), and myeloperoxidase-1 (MPO-1) (Jiancheng Bioengineering Co., LTD., Nanjing, China) by colorimetry.

#### 2.7.4. Assessment of Endothelial Active Substances

ELISA (3001, Thermo, Waltham, MA, USA) was used to detect the contents of nitric oxide (NO) (Jiancheng Bioengineering Co., LTD., China) and endothelin-1 (ET-1) (Beinglay Biotechnology, Wuhan, China) in serum by ultraviolet spectrophotometry (UV-2600, Shimadzu, Kyoto, Japan) was used to determine the activity of serum nitric oxide synthase (NOS) (Jiancheng Bioengineering Co., LTD., Nanjing, China).

### 2.8. Histopathological Examination

Seven days after modeling, the left ventricular myocardial tissue was immediately taken while blood was drawn from the abdominal aorta. After washing the tissue three times with phosphate-buffered saline (PBS), fixed with 4% paraformaldehyde (pH 7.4), embedded in conventional paraffin and sectioned (DB9, Delisen, Hubei, China), the sections were dewaxed with conventional xylene, graded with ethanol, and rinsed with water. Then, hematoxylin-eosin (H&E) staining, conventional dehydration, transparency, and resin sealing were used to observe the pathological changes of the myocardium.

### 2.9. Detection of Apoptosis by the TUNEL Assay

Repeating the steps above, deoxynucleoside transferase was catalyzed by adding fluorescein-labeled deoxyuridine triphosphate and apoptosis was observed under a 400× light microscope (CKX41, Olympus, Tokyo, Japan). Six different fields of vision were randomly selected for each slice to calculate the myocardial cell apoptosis rate. The calculation formula was as follows:

(1) $$\mathrm{Myocardial}\;\mathrm{cell}\;\mathrm{apoptosis}\;\mathrm{rate}=\frac{\mathrm{Apoptotic}\;\mathrm{cardiomyocytes}}{\mathrm{Total}\;\mathrm{myocardial}\;\mathrm{cells}\;}\times 100\%.$$

### 2.10. Immunohistochemical Staining

For immunohistochemical staining, the steps of H&E staining as outlined above were followed, and the tissues were blocked with goat serum at room temperature for 30 min, and incubated with primary antibody for SIRT3 and AMPK at 4 °C overnight. The next day, after being washed with PBS, the tissues were incubated with HRP-labeled secondary antibody in the dark at room temperature for 60 min. After diaminobenzidine staining, hematoxylin was used for counterstaining. The stained sections were observed under a light microscope, and the images were analyzed with Image J software (WS Rasband, NIH, http://imagej.nih.gov/ij/).

### 2.11. Real-Time Polymerase Chain Reaction (PCR) Analysis

The total RNA was extracted with an extraction kit after the cardiac tissue in the apex was chopped and milled, and the purity and integrity of the RNA were detected by agarose gel electrophoresis. The sample with absorbance A_260_/A_280_ ≥ 1.80 was qualified. cDNA was synthesized by reverse transcription from the diluted RNA sample for PCR (LC480IIRT-PCR, Roche, Swiss). The primer sequences were as follows: β-actin (189 bp) upstream primer 5′-CCACCATGTACCCAGGCATT-3′, downstream primer 5′-CGGACTCATCGTACTCCTGC-3’; Bcl-2 (90 bp) upstream primer 5′-GATTGTGGCCTTCTTTGAGT-3′, downstream primer 5′-CACAGAGCGATGTTGTCC-3’; Bax (85 bp), upstream primer 5′-TGAGCTGACCTTGGAGCA-3′, downstream primer 5’-GTCCAGTTCATCGCCAAT-3′. The PCR program was as follows: 95 °C for 5 min, 95 °C for 10 s, 60 °C for 30 s for 40 cycles. β-actin was used as an internal reference, and the relative expression of mRNA was calculated according to the 2^−ΔΔCt^ method.

### 2.12. Western Blotting Analysis

Cardiac tissue cells were collected in RIPA buffer containing protease inhibitor. After centrifugation, the concentration of total protein in the supernatant was determined with a BCA protein detection kit. Total protein (10–100 µg) was separated by 10% SDS-PAGE electrophoresis and then transferred to polyvinylidene fluoride membranes (PVDF). After being sealed in PBS containing 5% skim milk powder, a primary antibody was added and incubated overnight at 4 °C. After incubating with the secondary antibody labeled with HRP for 2 h, the membranes were washed with PBS containing 0.1% Tween-20. PVDF were detected and quantified, and repeated three times to determine the average.

### 2.13. Statistical Analysis

SPSS20.0 software (ND Times Technology Co., LTD., Beijing, China) was used for statistical analysis. The experimental results were expressed as the mean ± standard deviation of at least three independent samples. Based on the assumption that the data showed a normal distribution, a one-way analysis of variance was performed to compare the differences between the groups. *p* < 0.05 was considered statistically significant.

## 3. Results

### 3.1. Characterization of Nanoparticles

**Figure 1:**
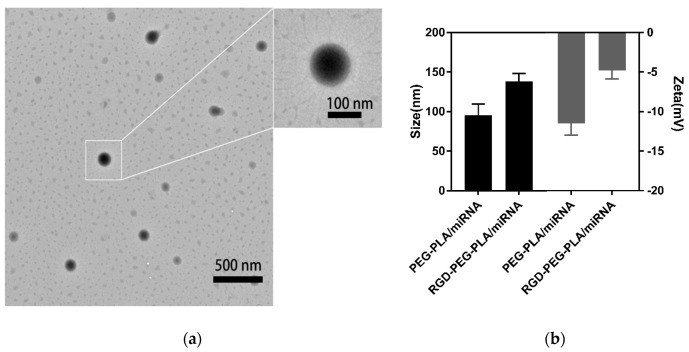
Characterization of nanoparticles. (**a**) Transmission electron microscopy (TEM) image of arginine-glycine-aspartic acid tripeptide (RGD)-polyethylene glycol (PEG)-polylactic acid (PLA)/ microRNA-133 (miR-133). (**b**) The average particle size and surface zeta potential of PEG-PLA/miR-133 and RGD-PEG-PLA/miR-133.

The TEM image (Figure 1a) showed that the prepared nanoparticles had a spherical morphology. The particle size and zeta potential of the nanoparticles were measured by a Malvern particle size analyzer (Supplementary Materials Table S1). As Figure 1b, the average particle size of RGD-PEG-PLA/miR-133 was approximately 137.9 nm, the average surface zeta potential of RGD-PEG-PLA/miR-133 was approximately −4.9 mV, and the polydispersity index obtained was less than 0.24. The nanoparticle miRNA encapsulation efficiency was 80.1% (5 nmol miRNA/per mg nanoparticles). Nanoparticles were stable in aqueous solution with good uniformity.

### 3.2. Biodistribution of RGD-PEG-PLA Nanoparticles In Vivo

**Figure 2:**
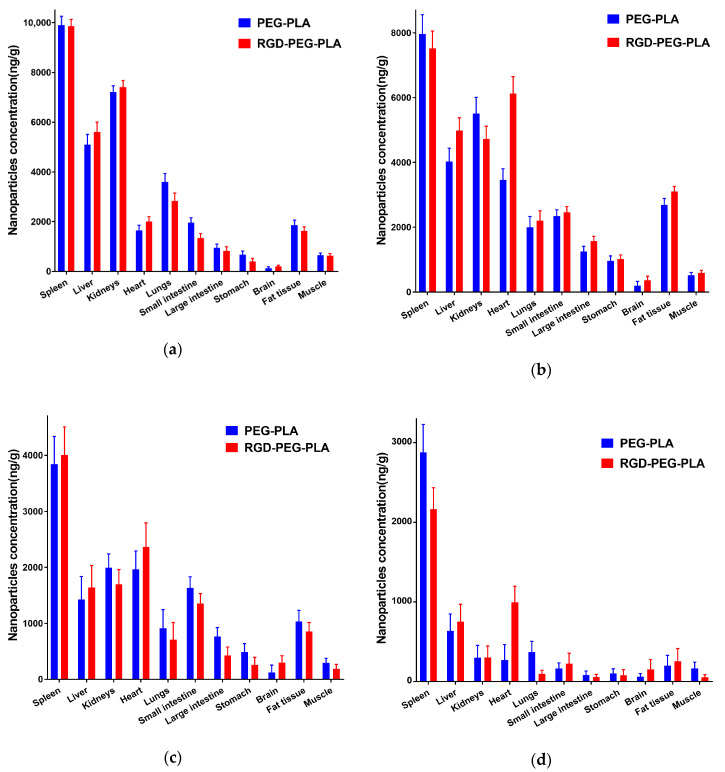
Concentration of nanoparticles in the spleen, liver, kidneys, heart, lungs, small intestine, large intestine, stomach, brain, fat tissue, and muscle after administration of PEG-PLA and RGD-PEG-PLA in rat models at (**a**) 0.5 h, (**b**) 4 h, (**c**) 12 h, (**d**) 36 h (mean ± standard deviation (SD), *n* = 6).

**Figure 3:**
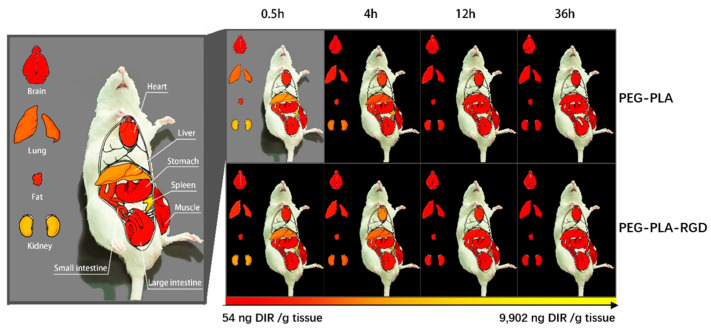
The specific biodistribution of nanoparticles in rats, the distribution of non-targeted PEG-PLA/1,1-dioctadecyl-3,3,3,3-tetramethylindotricarbocyaine iodide (DIR) and RGD-PEG-PLA/DIR nanoparticles varies by organs and tissues.

To verify whether RGD-modified nanoparticles can effectively deliver miRNA to the heart, we analyzed DIR delivery by injecting nanoparticles into the caudal vein of rats. At 0.5 h, significant DIR enrichment was first detected in the RGD-PEG-PLA and PEG-PLA groups in the kidney, liver, lungs, and spleen of the rats (Figure 2 and Figure 3). The maximum DIR level (9902 ng/g) was detected in the spleen delivered by the PEG-PLA group, with RGD-PEG-PLA having a lower DIR level (9861 ng/g). DIR concentrations decreased significantly over time in the kidneys, liver, and lungs in both groups. After 4 h, RGD-PEG-PLA delivery DIR was significantly enriched in the heart, 1.8 times higher than the PEG-PLA group. There was no significant difference in delivery between RGD and unmodified nanoparticles in any peripheral organ or tissue. In addition, the RGD-PEG-PLA group had a long retention time in the heart. After 36 h, the level was still enriched at 996 ng/g in the heart, while the concentration without RDG modification was reduced to 268 ng/g. The visualization experiment results showed that the RGD-modified nanoparticles maintained a high distribution in the heart within 4–24 h, second only to the spleen, liver, and kidneys, indicating that the RGD-modified nanoparticles could accumulate more in the heart.

### 3.3. Effects of RGD-PEG-PLA/miR-133 on the Electrocardiogram of AMI Rats

**Figure 4:**
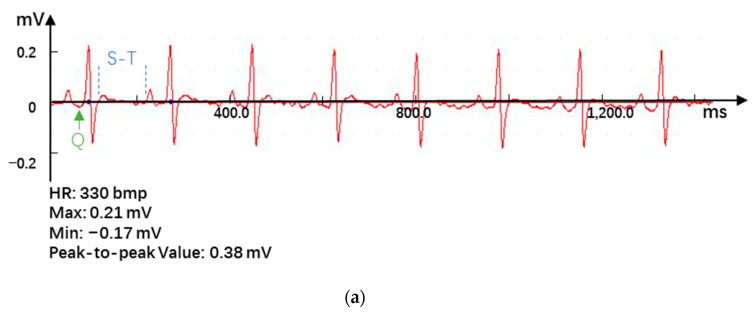

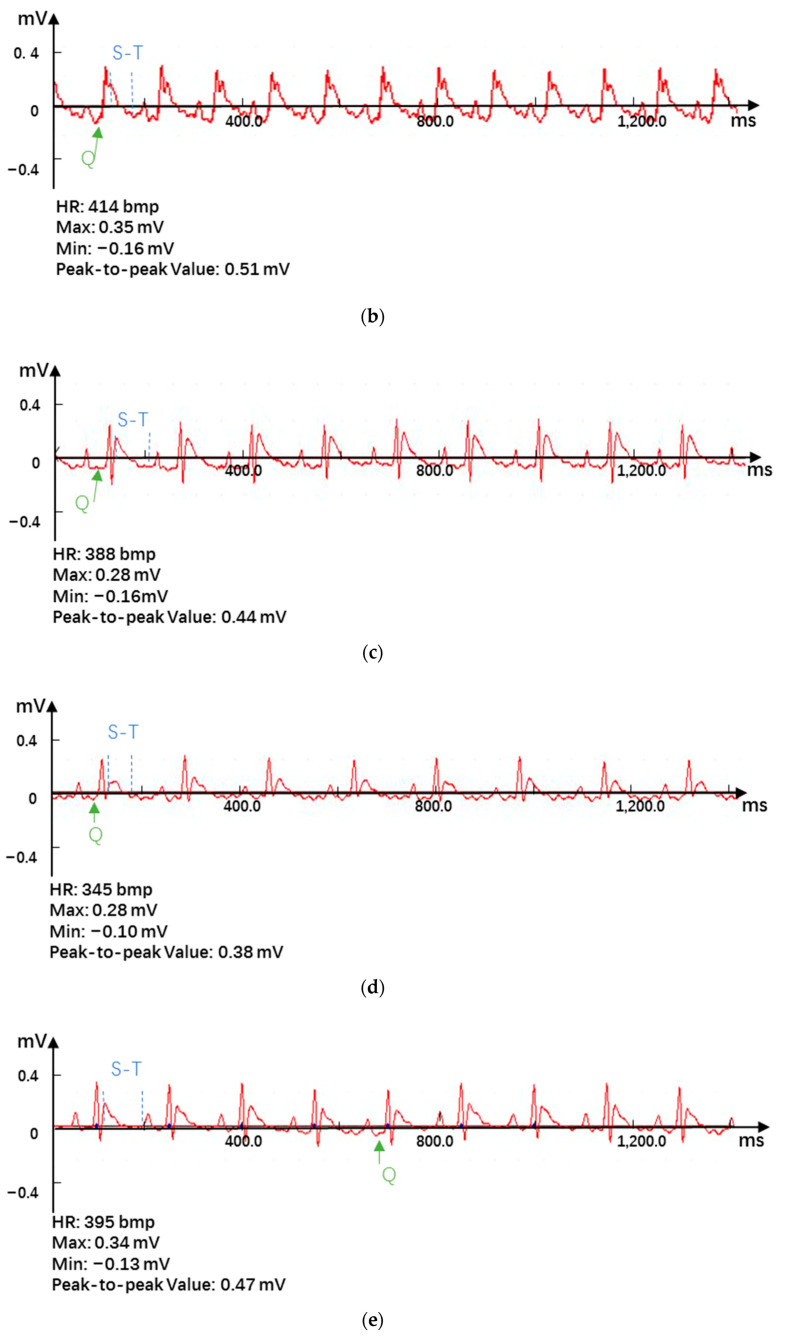

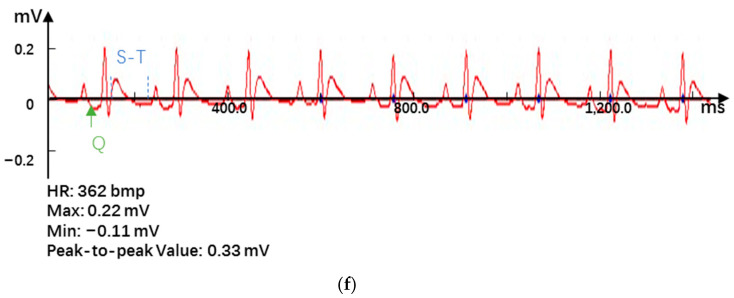
Electrocardiogram results in (**a**) the sham group, (**b**) the model group, (**c**) the positive drug group, (**d**) the miR-133 group, (**e**) the PEG-PLA/miR-133 group, and (**f**) the RGD-PEG-PLA/miR-133 group of rats.

The significant changes in the ECG (Supplementary Materials Table S2) when AMI occurred included the marked increase in the ST segment and the appearance of pathological Q waves [3,9]. The results showed that after ligation of the LAD, the ECG showed obvious waveform changes, the ST segment elevation was obvious, and the heart rate was significantly accelerated, indicating that the AMI model was successfully established (Figure 4a,b). Accordingly, the consistency of the degree of AMI in each group can be evaluated. After administration, the ST segment of the ECG was significantly reduced, especially in the positive drug group and the RGD-PEG-PLA/miR-133 group (Figure 4c–f).

### 3.4. Effects of RGD-PEG-PLA/miR-133 on the Echocardiography of AMI Rats

#### 3.4.1. Effects of RGD-PEG-PLA/miR-133 on the Left Ventricle Morphology in AMI Rats

**Figure 5:**
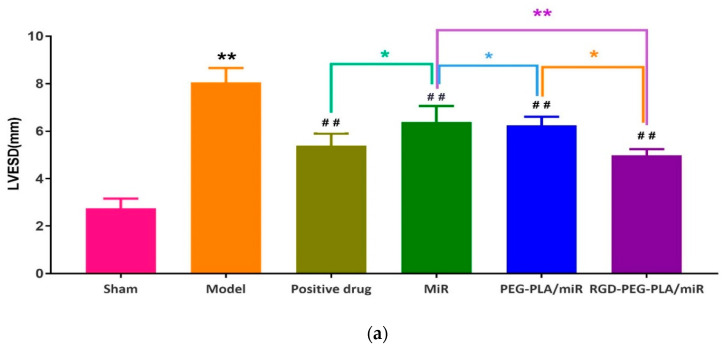

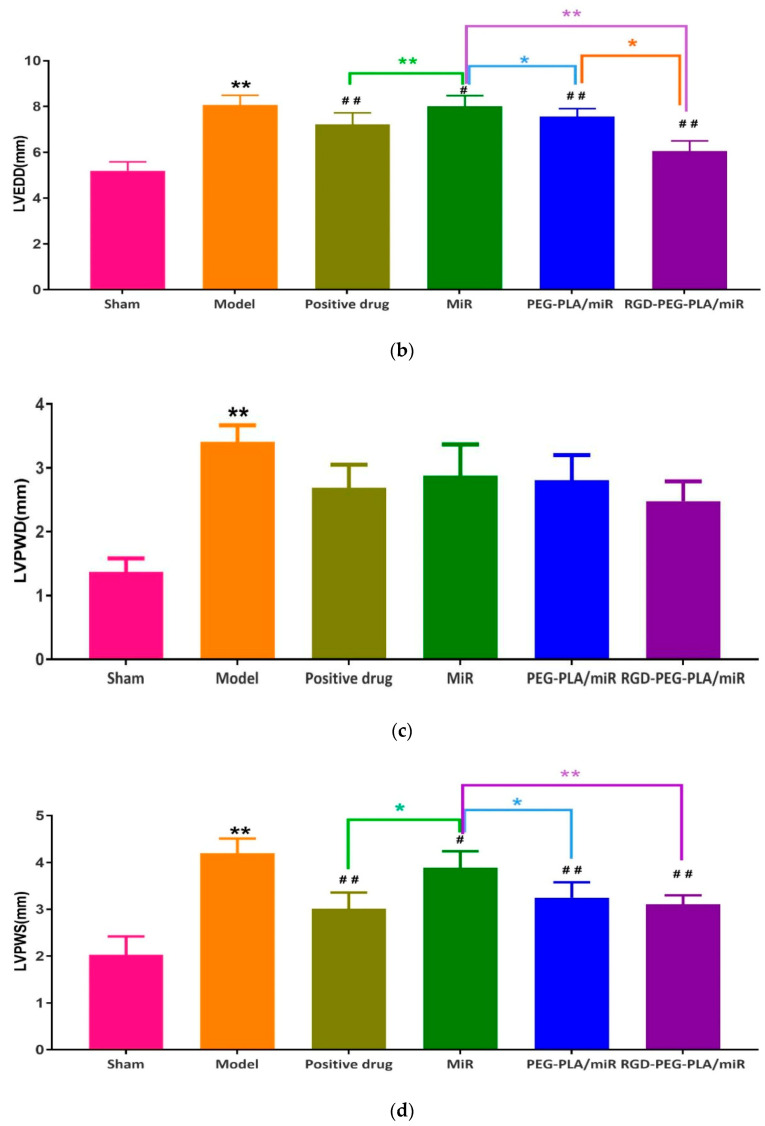
Results of (**a**) left ventricular end-systolic diameter (LVESD), (**b**) left ventricular end-diastolic diameter (LVEDD), (**c**) left ventricular posterior wall thickness at end-diastole (LVPWD), and (**d**) left ventricular posterior wall thickness at end-systole (LVPWS) on echocardiography in six groups of rats (mean ± SD, *n* = 6). Compared with the sham group, the difference was statistically significant (** *p* < 0.01). Compared with the model group, the difference was statistically significant (^#^ *p* < 0.05, ^##^ *p* < 0.01). The color asterisks indicate significant differences between the two groups, * *p* < 0.05 and ** *p* < 0.01.

LVESD, LVEDD, LVPWD, and LVPWS are the indexes to evaluate left ventricle morphology. Compared with those of the sham group, the LVESD, LVEDD, LVPWD, and LVPWS of the model group were significantly higher (*p* < 0.01), as shown in Figure 5. The left ventricular cavity was enlarged and the anterior wall of the left ventricle was thickened, which indicated that the morphological structure of the heart changes after AMI in rats, and the modeling was successful.

Compared with those of the model group, the LVESD, LVEDD, and LVPWS of the administered groups were significantly lower. The LVPWD of the administered groups was slightly lower, and this difference was not significant. Compared with those in the miR-133 group, the LVESD, LVEDD, and LVPWS in the positive drug group, the PEG-PLA/miR-133 group, and the RGD-PEG-PLA/miR-133 group were significantly lower. LVEDD was significantly different between the PEG-PLA/miR-133 group and the RGD-PEG-PLA/miR-133 group (*p* < 0.05).

#### 3.4.2. Effects of RGD-PEG-PLA/miR-133 on the Left Ventricular Function in AMI Rats

**Figure 6:**
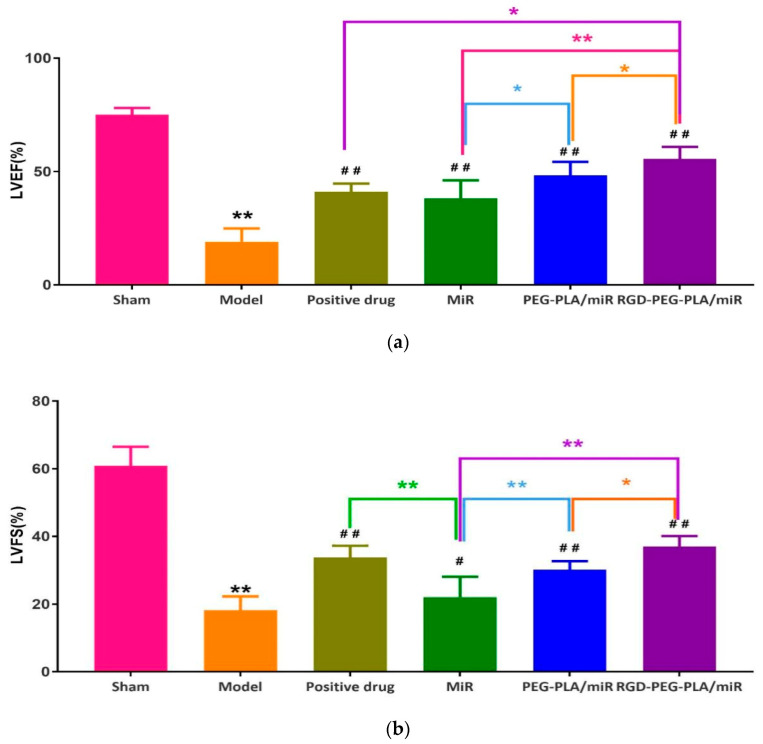
Results of (**a**) left ventricular ejection fraction (LVEF) and (**b**) left ventricular fraction shortening (LVFS) on echocardiography in six groups of rats (mean ± SD, *n* = 6). Compared with the sham group, the difference was statistically significant (** *p* < 0.01). Compared with the model group, the difference was statistically significant (^#^ *p* < 0.05, ^##^ *p* < 0.01). The color asterisks indicate significant differences between the two groups, * *p* < 0.05 and ** *p* < 0.01.

LVEF and LVFS are the main indexes to evaluate left ventricular function. LVEF reflects the shortening ability of left ventricular myocardial fiber, while LVFS reflects the relationship between stress and shortening. As can be seen from Figure 6, compared with those of the sham group, the LVEF and LVFS of the model group were significantly lower (*p* < 0.01). It indicated that the cardiac ejection function of rats decreased after AMI, and the modeling was successful. Compared with those of the model group, the LVEF and LVFS of the administered groups were significantly higher. Compared with the miR-133 group, the PEG-PLA/miR-133 group, the RGD-PEG-PLA/miR-133 group, and the positive drug group had more significant effects on LVEF and LVFS.

### 3.5. Effects of RGD-PEG-PLA/miR-133 on the Biochemical Indicators of AMI Rats

#### 3.5.1. Effects of RGD-PEG-PLA/miR-133 on Cardiac-Specific Markers

**Figure 7:**
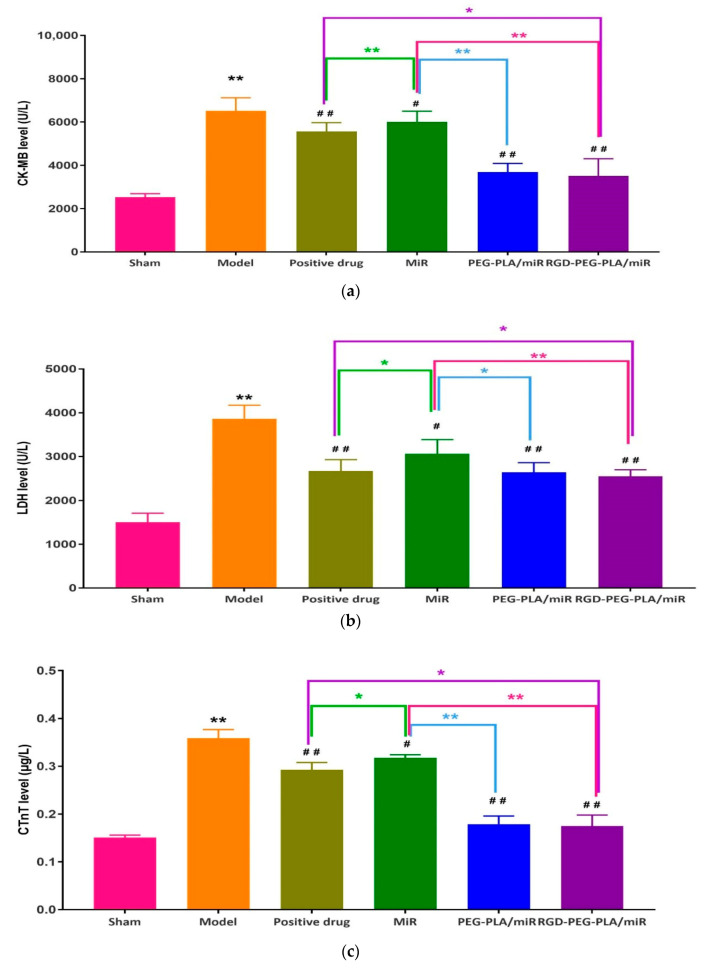
The levels of (**a**) creatine kinase isoenzyme (CK-MB), (**b**) lactic dehydrogenase (LDH), and (**c**) cardiac troponin T (cTnT) in six groups of rats (mean ± SD, *n* = 6). Compared with the sham group, the difference was statistically significant (** *p* < 0.01). Compared with the model group, the difference was statistically significant (^#^ *p* < 0.05, ^##^ *p* < 0.01). The color asterisks indicate significant differences between the two groups, * *p* < 0.05 and ** *p* < 0.01.

Cardiac-specific markers, namely cTnT, CK-MB, and LDH, were used to evaluate the apoptosis of myocardial cells mediated by mitochondrial injury [21]. From Figure 7, we can see that compared with those in the sham group, the levels of CK-MB, LDH, and cTnT in the model group were significantly higher (*p* < 0.01). Compared with those in the model group, the levels of CK-MB, LDH, and cTnT in the administered groups were significantly lower. Compared with those in the miR-133 group, the levels of CK-MB and cTnT in the PEG-PLA/miR-133 group, the RGD-PEG-PLA/miR-133 group, and the positive drug group were significantly lower, especially in the RGD-PEG-PLA/miR-133 group (*p* < 0.01). The serum levels of myocardial injury markers in AMI rats was significantly increased, and the levels were significantly decreased after administration, suggesting that miR-133 could alleviate myocardial injury in AMI rats.

#### 3.5.2. Effects of RGD-PEG-PLA/miR-133 on Oxidative Stress Markers

**Figure 8:**
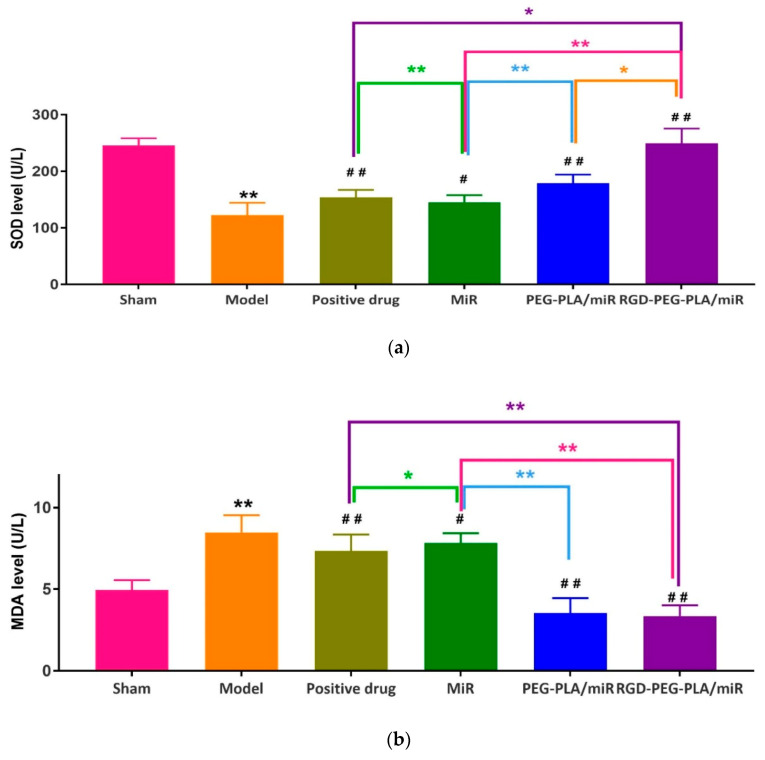

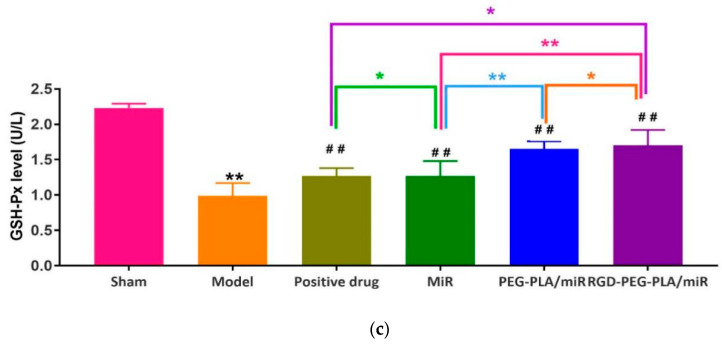
The levels of (**a**) superoxide dismutase (SOD), (**b**) malondialdehyde (MDA), and(**c**) glutathione peroxidase (GSH-Px) in six groups of rats (mean ± SD, *n* = 6). Compared with the sham group, the difference was statistically significant (** *p* < 0.01). Compared with the model group, the difference was statistically significant (^#^ *p* < 0.05, ^##^ *p* < 0.01). The color asterisks indicate significant differences between the two groups, * *p* < 0.05 and ** *p* < 0.01.

The enhanced oxidative stress has been confirmed to be a key contributor to increased myocardial injury after acute myocardial infarction. The mitochondrial oxidative stress markers (SOD activity, MDA content, GSH-Px activity) evaluated oxidative-stress-induced cellular apoptosis [17]. As can be seen from Figure 8, compared with those in the sham group, the activities of SOD and GSH-Px in the model group were significantly lower, and the level of MDA was significantly higher (*p* < 0.01). Compared with the model group, the administered groups had significantly higher SOD and GSH-Px activities and lower MDA levels. Compared with the miR-133 group, the PEG-PLA/miR-133 group, the RGD-PEG-PLA/miR-133 group, and the positive drug group showed significantly higher SOD and GSH-Px activities and lower MDA levels (*p* < 0.01). The results indicated that the redox status of the serum following myocardial infarction was improved by administration.

#### 3.5.3. Effects of RGD-PEG-PLA/miR-133 on Serum Inflammatory Cytokines

**Figure 9:**
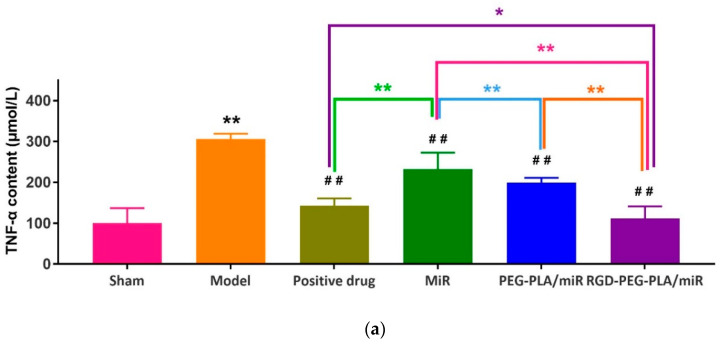

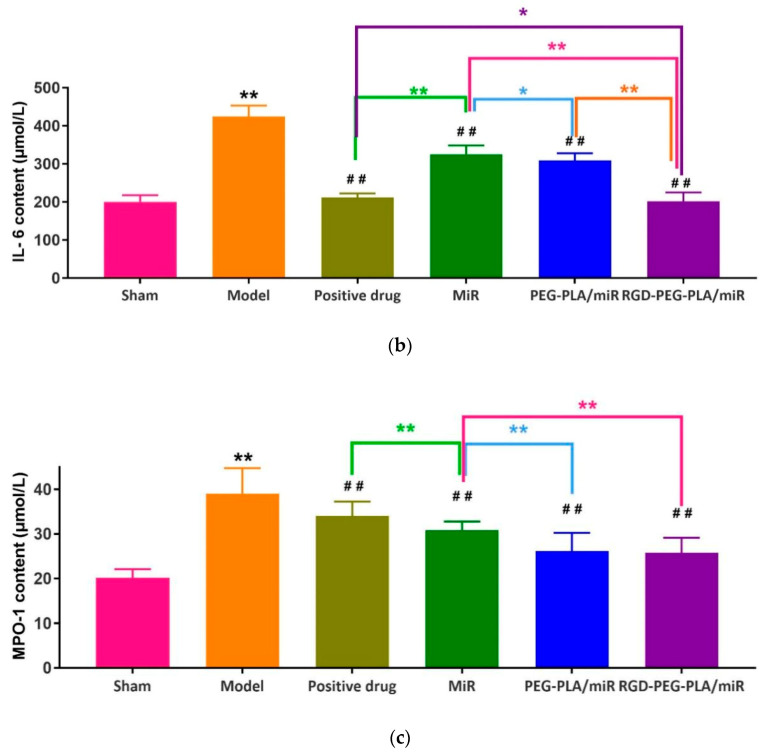
The contents of (**a**) tumor necrosis factor-α (TNF-α), (**b**) interleukin-6 (IL-6), and (**c**) myeloperoxidase-1 (MPO-1) in six groups of rats (mean ± SD, *n* = 6). Compared with the sham group, the difference was statistically significant (** *p* < 0.01). Compared with the model group, the difference was statistically significant (^##^ *p* < 0.01). The color asterisks indicate significant differences between the two groups, * *p* < 0.05 and ** *p* < 0.01.

The inflammatory reaction of myocardial cells can be assessed by detecting changes in TNF-α, IL-6, and MPO-1 serum contents in AMI model rats. As seen in Figure 9, compared with those in the sham group, the contents of TNF-α, IL-6, and MPO-1 in the model group were significantly higher (*p* < 0.01). Compared with those in the model group, the contents of TNF-α, IL-6, and MPO-1 in the administered groups were significantly lower. Compared with those in the miR-133 group, the contents of TNF-α, IL-6, and MPO-1 in the PEG-PLA/miR-133 group, the RGD-PEG-PLA/miR-133 group, and the positive drug group were significantly lower (*p* < 0.01), especially in the RGD-PEG-PLA/miR-133 group. The results proved that miR-133 administration suppressed the inflammation following myocardial infarction.

#### 3.5.4. Effects of RGD-PEG-PLA/miR-133 on Endothelial Active Substances

**Figure 10:**
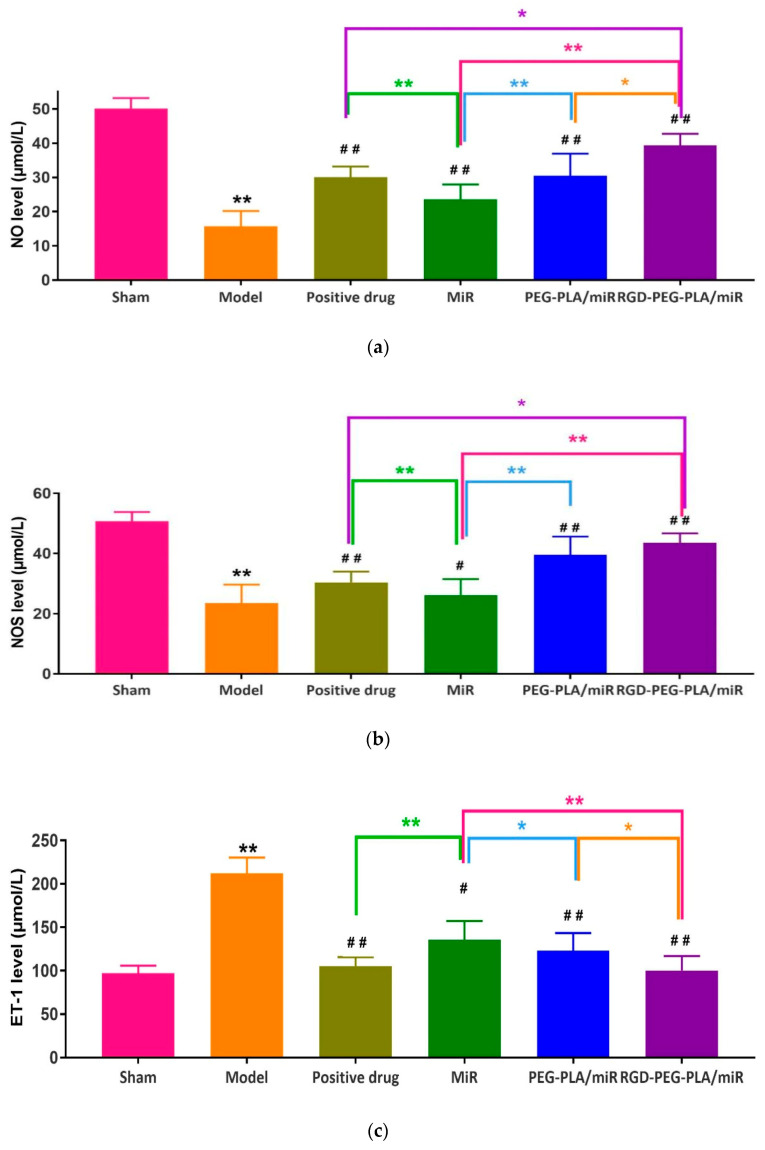
The levels of (**a**) nitric oxide (NO), (**b**) nitric oxide synthase (NOS), and (**c**) endothelin-1 (ET-1) in six groups of rats (mean ± SD, *n* = 6). Compared with the sham group, the difference was statistically significant (** *p* < 0.01). Compared with the model group, the difference was statistically significant (^#^ *p* < 0.05, ^##^ *p* < 0.01). The color asterisks indicate significant differences between the two groups, * *p* < 0.05 and ** *p* < 0.01.

The degree of vascular endothelial cell damage after myocardial infarction can be assessed by measuring the changes in NO, NOS, and ET-1 serum levels in AMI model rats. As seen in Figure 10, compared with those in the sham group, the NO and NOS expression levels in the model group were significantly lower, and ET-1 expression was significantly higher (*p* < 0.01). Compared with those in the model group, the expression levels of NO and NOS in the administered groups were significantly higher, and ET-1 expression was significantly lower. Compared with those in the miR-133 group, the expression levels of NO and NOS in the PEG-PLA/miR-133 group, the RGD-PEG-PLA/miR-133 group, and the positive drug group were significantly higher, and the expression of ET-1 was significantly lower (*p* < 0.01). It indicated that the increase of endothelial active substances induced by myocardial infarction could be inhibited after miR-133 administration.

### 3.6. Effects of RGD-PEG-PLA/miR-133 on the Histopathological Histology of AMI Rats

**Figure 11:**
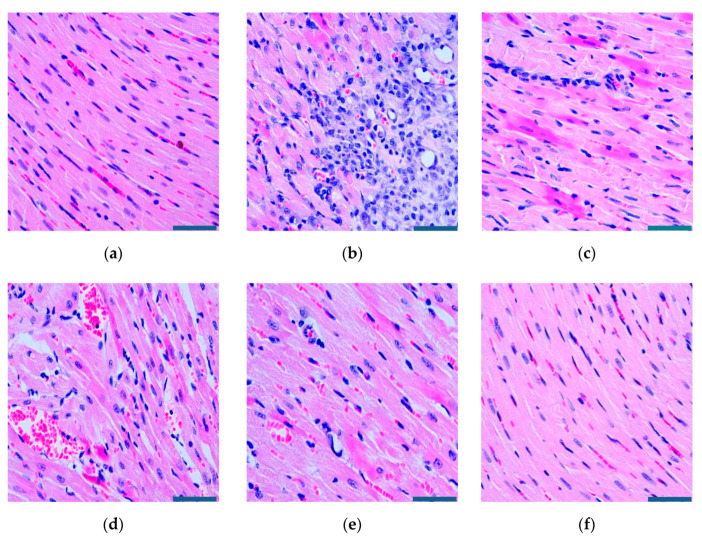
The myocardial histopathological representative changes of rats (×400, scale bar = 200 µm) in (**a**) the sham group, (**b**) the model group, (**c**) the positive drug group, (**d**) the miR-133 group, (**e**) the PEG-PLA/miR-133 group, and (**f**) the RGD-PEG-PLA/miR-133 group.

In the sham group, the volume and quality of the heart were the same as those of a normal heart, with a rosy color and no adhesion. As can be intuitively seen from Figure 11a, cardiomyocytes were arranged in an overall regular distribution with a clear cell structure, a centered nucleus, a uniform coloration, slight edema, and a small amount of inflammatory cell infiltration. In the model group, the left ventricular anterior wall infarction area was obviously gray or white and sagging, and the room wall was thinning. Figure 11b shows a disordered arrangement of myocardial cells, blurred stripes, degeneration, and necrosis in some areas, cell swelling, apoptosis with inflammatory infiltration, partial nuclear shrinkage and disappearance, and a large amount of fibrosis in the infarct area. Compared with the model group, H&E staining of hearts in the medication group showed that the arrangement of myocardial cells in the miR-133 group was still disordered, the stripes were blurred, the cells were still swollen, the vacuoles changed, and the inflammatory cells infiltrated. The myocardial cell disorder was significantly reduced in the positive drug group, the PEG-PLA/miR-133 group, and the RGD-PEG-PLA/miR-133 group. The tissue fiber coloration was relatively clear, the cells were slightly edematous, the striated part was broken, there was a few inflammatory cell infiltration, regional degeneration and necrosis were alleviated, a small amount of fibrosis changed in the infarct area, and red blood cells were significantly reduced.

### 3.7. Effects of RGD-PEG-PLA/miR-133 on Cardiomyocyte Apoptosis in AMI Rats

**Figure 12:**
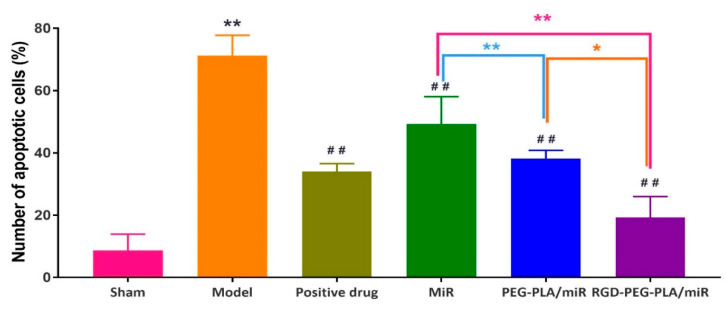
The number of apoptotic cardiomyocytes in six groups of rats (mean ± SD, *n* = 6). Compared with the sham group, the difference was statistically significant (** *p* < 0.01). Compared with the model group, the difference was statistically significant (^##^ *p* < 0.01). The color asterisks indicate significant differences between the two groups, * *p* < 0.05 and ** *p* < 0.01.

**Figure 13:**
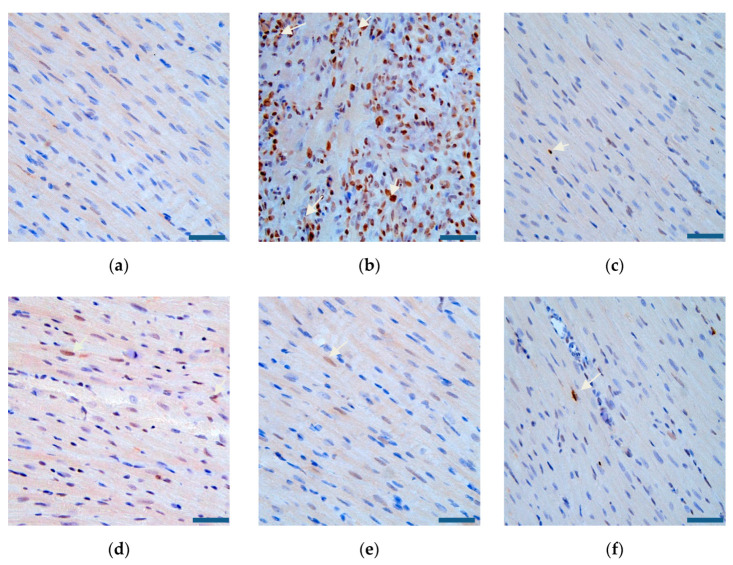
Cardiomyocyte apoptosis of rats (×400, scale bar = 200 µm) in (**a**) the sham group, (**b**) the model group, (**c**) the positive drug group, (**d**) the miR-133 group, (**e**) the PEG-PLA/miR-133 group, and (**f**) the RGD-PEG-PLA/miR-133 group. Blue: the nucleus of normal cells, brown: the nucleus of apoptotic cells. White arrows indicate the nucleus of apoptotic cells.

It can be seen from Figure 12 and Figure 13 that the myocardial tissue of the sham group was dominated by blue normal cells and that only some brown apoptotic cells were present. There were significantly more brown apoptotic cells in the myocardial tissue of the model group than in the sham group. Compared with that in the model group, the numbers of apoptotic cells in the myocardial tissue of the positive drug group and the miR-133 group were lower, and the difference was statistically significant.

### 3.8. Effects of RGD-PEG-PLA/miR-133 on the Expression of the SIRT3/AMPK Pathway in AMI Rats

**Figure 14:**
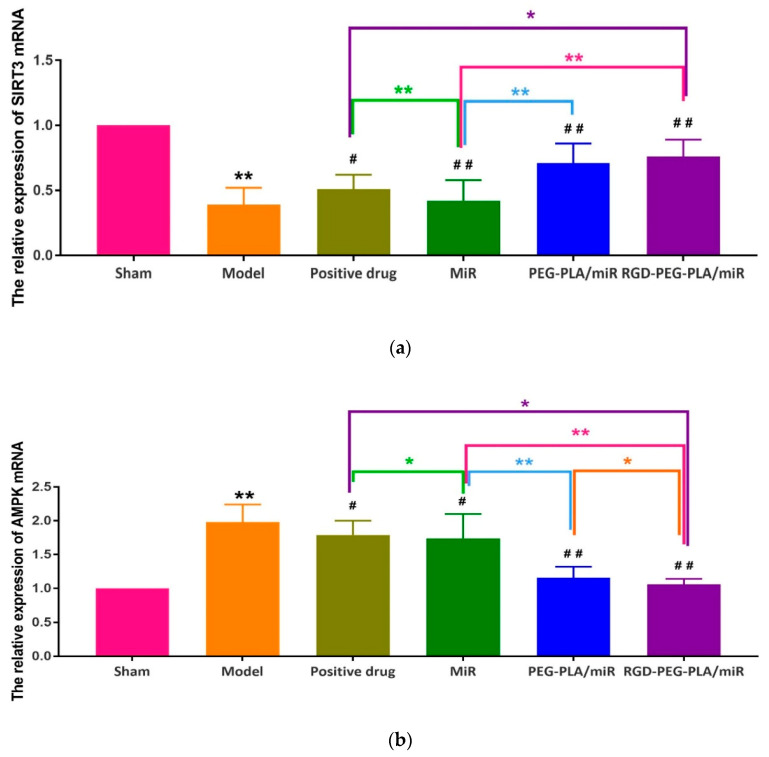
The relative expression of (**a**) Sirtuin3 (SIRT3) mRNA and (**b**) adenosine monophosphate activated protein kinase (AMPK) mRNA in six groups of rats (mean ± SD, *n* = 6). Compared with the sham group, the difference was statistically significant (** *p* < 0.01). Compared with the model group, the difference was statistically significant (^#^ *p* < 0.05, ^##^ *p* < 0.01). The color asterisks indicate significant differences between the two groups, * *p* < 0.05 and ** *p* < 0.01.

As Figure 14 shows, compared with that in the model group, the expression of SIRT3 mRNA in the positive drug group, the PEG-PLA/miR-133 group, and the RGD-PEG-PLA/miR-133 group was significantly higher, and the expression of AMPK mRNA was significantly lower (*p* < 0.05, *p* < 0.01). Compared with that in the miR-133 group, SIRT3 mRNA expression increased and AMPK mRNA expression decreased most significantly in the PEG-PLA/miR-133 group, the RGD-PEG-PLA/miR-133 group, and the positive drug group (*p* < 0.01).

**Figure 15:**
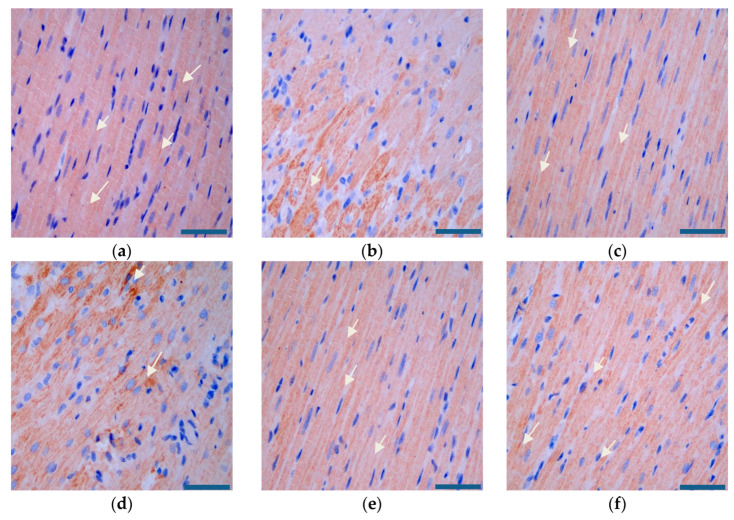
SIRT3 expression in the rat heart tissues (×400, scale bar = 200 µm) of (**a**) the sham group, (**b**) the model group, (**c**) the positive drug group, (**d**) the miR-133 group, (**e**) the PEG-PLA/miR-133 group, and (**f**) the RGD-PEG-PLA/miR-133 group. Blue: nucleus, brown: SIRT3-positive stain. White arrows indicate the regions of strongly positive SIRT3 expression.

**Figure 16:**
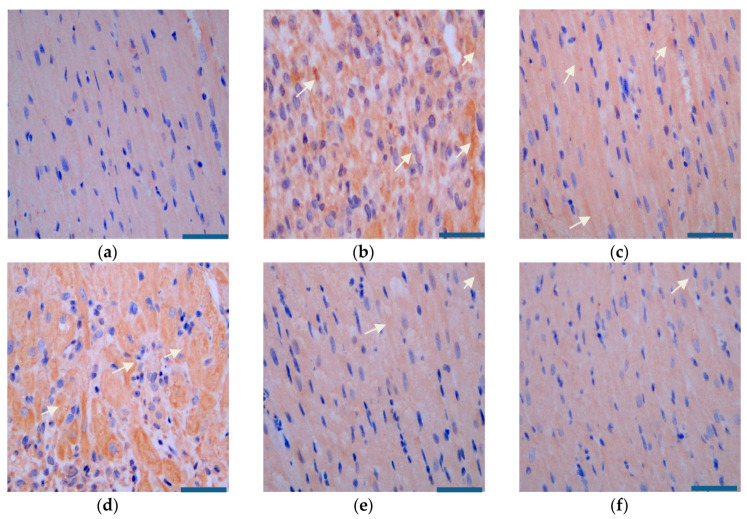
AMPK expression in the rat heart tissues (×400, scale bar = 200 µm) of (**a**) the sham group, (**b**) the model group, (**c**) the positive drug group, (**d**) the miR-133 group, (**e**) the PEG-PLA/miR-133 group, and (**f**) the RGD-PEG-PLA/miR-133 group. Blue: nucleus, brown: AMPK-positive stain. White arrows indicate the regions of strongly positive AMPK expression.

As Figure 15 and Figure 16 show, compared with that in the model group, the expression of SIRT3 was significantly higher and the expression of AMPK was significantly lower in the administered groups. Compared with that in the miR-133 group, the expression of SIRT3 increased and the expression of AMPK decreased most significantly in the PEG-PLA/miR-133 group, the RGD-PEG-PLA/miR-133 group, and the positive drug group. 

## 4. Discussion

In the experiment, the blood supply to the heart from the left coronary artery of the rat was completely blocked. According to the occurrence of myocardial infarction, the ST segment increased significantly, and pathological Q waves appeared on the ECG. The success of the disease model construction can be identified qualitatively, and the consistency of the initial conditions of the models in each group can be evaluated [30]. Adopting the visualization method, we detected fluorescent tags with RGD-modified PEG-PLA nanoparticle and non-RGD-modified PEG-PLA nanoparticle distributions in the models and deduced the RGD-PEG-PLA nanoparticle delivery of biodistribution in vivo. The results intuitively showed that the RGD modification gave the nanoparticles the ability to aggregate in the heart. In addition, the nanoparticles were also distributed in large amounts to the liver, kidneys, spleen, and other high-perfusion rate organs. Therefore, we selected miR-133, which is mainly specifically expressed in myocardial and skeletal muscle tissues, to play a therapeutic role in AMI [11,17,30,31]. After intravenous administration, the nanoparticles are less distributed in the skeletal muscle tissue and are mainly distributed in organs such as heart, liver, spleen, and kidneys. Targeted delivery vehicles can allow more miRNAs to be trapped in the myocardial infarction area and in action. To some extent, it overcomes the limitations of miRNA in the field of treatment, which is difficult to penetrate biological barriers in vivo to reach the site of action, and rapidly degrade and clear in the blood circulation [18]. However, the use of miR-21, which has the same cardioprotective effect, has the potential risk of promoting liver and kidney fibrosis [6]. It is mainly highly expressed in the liver and kidney. According to the different administration in the experiment, the rats were divided into the sham group, the model group, the positive drug group, the miR-133 group, the PEG-PLA/miR-133 group, and the RGD-PEG-PLA/miR-133 group. The design used random control procedures, a single variable and the parallel repetition principle, and aspirin was used as a positive control drug. After administration, the changes in the related indexes of rats in each group were detected from different perspectives to evaluate the therapeutic effect. In cardiac functional studies, both electrocardiography and echocardiography were used to evaluate the therapeutic effect of miR-133 in vivo by observing the changes in the electrocardiogram with respect to the LVEDD, LVESD, LVPWD, LVPWS, LVEF, and LVFS. Left ventricular morphology and function were determined, and the cardioprotective effect of RGD-PEG-PLA/miR-133 on AMI model rats was evaluated [3,5,12,32].

The occurrence and development of AMI is related to the occurrence of the inflammatory response, vascular endothelial injury, oxidative stress, metabolic disorders, mitochondrial dysfunction, and so on. Typical cardiac markers such as cTnT, CK-MB, and LDH have been clinically used for the early diagnosis and postmortem detection of AMI [2]. When AMI occurs, the inflammatory reaction leads to the increase of serum inflammatory cytokines TNF-α, IL-6, MPO-1 levels. Moreover, it further leads to vascular endothelial cell injury, which leads to increased ET-1 levels and decreased NO levels of endothelial active substances, causing platelet adhesion and aggregation and aggravating MIRI injury. Under oxidative stress, SOD compensation in the myocardial infarction area increases and can specifically eliminate oxygen free radicals in the body [4], thus reducing the damage of oxidative stress to myocardial cells. MDA indirectly reflects the severity of the free radical attack and the damage of myocardial cells by reflecting the level of free radicals in the body. In this study, we found that miR-133 inhibited oxidative stress by downregulating oxidative stress markers and endothelial active substances to reduce myocardial damage (Figure 8 and Figure 10), which was consistent with the results of previous studies [16]. MiR-133 may also protect myocardial cells by downregulating inflammatory factors TNF-α, IL-6, and MPO-1, reducing inflammatory responses (Figure 9), and may reduce myocardial cell damage by inhibiting myocardial injury markers (Figure 7). Histopathological and H&E results were consistent with this conclusion. The apoptosis of myocardial cells in the three groups treated with miR-133 was reduced (Figure 13), but RGD-PEG-PLA/miR-133 performed best, which may reflect the advantage of targeted delivery of miRNA by nanoparticles. In the H&E result, the myocardial cell arrangement disorder in the RGD-PEG-PLA/miR-133 group was significantly lower than that in the model group. There was only a small amount of inflammatory cell infiltration, regional degeneration, and necrosis were alleviated, a small number of fibrosis was observed in the infarction area, and there were significantly fewer red blood cells. The protective effect of RGD-PEG-PLA/miR-133 on cardiomyocytes of AMI rats was confirmed.

Studies have shown that SIRT3 plays a protective role in cardiovascular diseases [33]. SIRT3 protein is a mitochondrial deacetylase that is abundant in the heart, liver, and other tissues. It regulates mitochondrial function, glucose and lipid metabolism, oxidative stress, and cell apoptosis through reversible lysine deacetylation [31]. AMPK is an energy state sensor that regulates various pathological responses of cells, including hypoxic ischemia and oxidative stress, and participates in many metabolic processes related to SIRT3 [33]. Previous studies have shown that miR-133 plays a cardioprotective role by mediating the signaling pathway associated with AMPK. For example, Zhang et al. showed that tanshinone IIA improved the expression of miR-133 in hypoxic-ischemic cardiomyocytes through the mitogen activated protein kinase (MAPK)-extracellular signal regulated kinase (ERK) 1/2 pathway [34]. Wang et al. stated that the MAPK/ERK signaling pathway of nicotine-induced cardiomyocyte apoptosis downregulated miR-133 [35]. Some scholars have directly confirmed that the AMPK 3’ untranslated regions (UTR) is a direct target of miR-133a [36]. From the perspective of mechanism, the protective effect of miR-133 on the heart is related to AMPK. AMPK and SIRT3 often regulate the same metabolic pathway, so the mechanism of action of miR-133 may also be related to SIRT3. From the perspective of the disease model, scholars have confirmed that SIRT3 is significantly reduced in the ischemic tissue of the rat model of coronary artery occlusion [37]. The AMI model in the experiment was also constructed from the blocked coronary artery, which is more suitable for the analysis of the relationship between miR-133 and SIRT3. Based on this, we hypothesized that miR-133 might exert a protective effect on the heart by affecting the SIRT3/AMPK pathway, and we conducted experiments to verify our hypothesis. The expression of mitochondrial SIRT3 and AMPK in myocardial tissue was observed by immunohistochemistry, and the expression levels of SIRT3 and AMPK mRNA were observed by PCR to explore whether the protective mechanism of miR-133 on myocardial tissue in AMI rats was related to the SIRT3/AMPK pathway. The results showed that the expression level of SIRT3 protein was significantly higher and the level of AMPK was significantly lower in the myocardial tissue of the RGD-PEG-PLA/miR-133 group than that in the model group. The protective effect of RGD-PEG-PLA/miR-133 nanoparticles on myocardial tissue of AMI rats may be achieved by upregulating SIRT3 and inhibiting AMPK in mitochondria. The mechanism may be related to the regulation of the SIRT3/AMPK pathway. However, miRNA-mediated gene regulation has the characteristics of multiple targets and multiple pathways, which means that miR-133 has more than a single target in the field of AMI. Further exploration is needed to reveal the molecular mechanism of miR-133. In addition, due to the limitations of setting up indicators and disease models in the experiment, there may be deviations due to the small number of samples. Therefore, the mechanism of miR-133 on AMI may be related to the regulation of the SIRT3/AMPK pathway. The changes in SIRT3 and AMPK expression levels in myocardial cells of AMI rats in the positive drug group may be caused by aspirin’s regulation of glucose and lipid metabolism in vivo and improvement of ischemia and hypoxia state of myocardial cells.

The main pathogenesis of myocardial infarction in the human body is coronary atherosclerosis, which causes the accumulation of lipids in the inner wall of the blood vessel, causing stenosis and even the occlusion of the lumen, and insufficient blood supply to the myocardium, which ultimately leads to the onset of AMI [7]. The AMI model prepared by coronary artery ligation only permanently blocked the left coronary artery of rats, which was actually an ischemia reperfusion model. This finding is not completely consistent with the pathological process of human myocardial infarction. However, since the onset of myocardial infarction is usually the result of coronary artery occlusion, this model is still widely used in animal experiments to simulate the incidence of AMI in humans [38]. The experimental study used coronary artery ligation to prepare the disease model, which is currently commonly used in an in vivo model of AMI [39]. Although the method is simple in principle, the modeling process is complicated, time-consuming, difficult, and has a low success rate. During modeling, many problems need to be solved. For example, the surgical process requires mechanical ventilation with an animal ventilator, the airway must be opened, and endotracheal intubation can easily cause adverse events, such as airway and esophageal injury. Due to the long operation time, the heart is exposed for a long time, and the wound surface is too large. These conditions are not conducive to the recovery of normal physiological functions. The use of thoracotomy is likely to damage other organs and tissues. Rib clipping damaged the normal anatomical structure of the rat’s chest and affected the recovery of spontaneous breathing. Special attention should be paid to keeping the rats warm and maintaining their body temperature. All of the above factors can reduce the survival rate of animal models. Therefore, the actual sample data obtained in the experiment are relatively limited. Moreover, the heart ischemic status of the disease model varies with time and individual differences, and the results may be random. The differences between some of the datasets may be smaller, and the changes may not be significant; thus, future research should use large samples.

In summary, the passive targeting of heart infarction lesions by the EPR effect of nanoscale carriers in the experiment was used to comprehensively investigate the biocompatibility and safety of nanomaterials. The nanoparticles composed of PEG-PLA were used as the carrier to modify RGD with the function of targeting heart infarction lesions [22,40]. In vivo biodistribution studies have shown that RGD-PEG-PLA can concentrate more in the heart and is an ideal carrier for miRNA delivery to the heart. The tail intravenous injection method was used to conduct examinations from multiple perspectives, including examinations of cardiac function, histopathology, hematology, and apoptosis, and a number of indicators were used to evaluate the effect of RGD-PEG-PLA nanoparticles containing miR-133 on AMI. The results showed that RGD-PEG-PLA/miR-133 could alleviate myocardial tissue damage to a certain extent, reduce cardiomyocyte apoptosis, and exert a cardiac protective effect by inhibiting the apoptosis, inflammatory response, and oxidative stress of myocardial cells in AMI rats. The mechanism may be related to the regulation of the SIRT3/AMPK pathway. RGD-PEG-PLA delivery resulted in increased accumulation of miR-133 in AMI diseased heart tissues, but how miR-133 binds to cardiomyocytes remains unclear, which may be an interesting topic for future research. The nanoparticles prepared by us may not only have great application potential in the field of cardiovascular diseases, but the strategy of active targeting of miR-133 to myocardial lesions for protective treatment may also provide enlightenment for the cardiotoxicity [41] induced by chemotherapy drugs. In addition, considering the potential toxicity of contrast agents [19], the nontoxic targeting nanocarrier can be used to deliver contrast agents for cardiac MRI.

## 5. Conclusions

This study is the first to combine active and passive targeting strategies for delivering miRNA to the heart, which is innovative. In vivo biodistribution studies have shown that RGD modification gives nanoparticles the ability to accumulate in cardiac infarction lesions, indicating that RGD-PEG-PLA can be an ideal carrier to target the heart. The delivery of miR-133 to the heart of AMI rats by the carrier can improve cardiac function damage, reduce myocardial infarction area, and exert cardiac protection by inhibiting apoptosis, inflammation, and oxidative stress. These effects may be related to the regulation of the SIRT3/AMPK pathway. RGD was used to modify the carrier, and the combination of RGD and the EPR effect of nanoparticles leads to higher levels of miR-133 in the myocardial infarction area [42], improves its bioavailability, and reduces its adverse effects on other target organs to a certain extent. These findings may provide a reference for the clinical application of miR-133.

## Supplementary Materials

The following are available online at https://www.mdpi.com/1999-4923/12/6/575/s1, Table S1: Size and zeta potential characterizations of different nanoparticles. Table S2. Typical changes of electrocardiogram in different groups.

## References

[1] Nicolini G., Forini F., Kusmic C., Pitto L., Mariani L., Iervasi G. (2015). Early and short-term triiodothyronine supplementation prevents adverse postischemic cardiac remodeling: Role of transforming growth factor-beta 1 and antifibrotic mirna signaling. Mol. Med..

[2] Pisano F., Altomare C., Cervio E., Barile L., Rocchetti M., Ciuffreda M.C., Malpasso G., Copes F., Mura M., Danieli P. (2015). Combination of miRNA499 and miRNA133 exerts a synergic effect on cardiac differentiation. Stem Cells.

[3] Zhang X.G., Wang L.Q., Guan H.L. (2019). Investigating the expression of miRNA-133 in animal models of myocardial infarction and its effect on cardiac function. Eur. Rev. Med. Pharmacol. Sci..

[4] Yu Y., Liu H., Yang D., He F., Yuan Y., Guo J., Hu J., Yu J., Yan X., Wang S. (2019). Aloe-emodin attenuates myocardial infarction and apoptosis via up-regulating miR-133 expression. Pharmacol. Res..

[5] Song Y., Zhang C., Zhang J., Jiao Z., Dong N., Wang G., Wang Z., Wang L. (2019). Localized injection of miRNA-21-enriched extracellular vesicles effectively restores cardiac function after myocardial infarction. Theranostics.

[6] Bejerano T., Etzion S., Elyagon S., Etzion Y., Cohen S. (2018). Nanoparticle delivery of miRNA-21 mimic to cardiac macrophages improves myocardial remodeling after myocardial infarction. Nano Lett..

[7] Wang S., Jiang J., Wang Y., Jia Q., Dai S., Wang Y., Lv L., Wang J. (2018). rLj-RGD3, a novel recombinant toxin protein from Lampetra japonica, prevents coronary thrombosis-induced acute myocardial infarction by inhibiting platelet functions in rats. Biochem. Biophys. Res. Commun..

[8] Goldbergova M.P., Lipkova J., Fedorko J., Sevcikova J., Parenica J., Spinar J., Masarik M., Vasku A. (2018). MicroRNAs in pathophysiology of acute myocardial infarction and cardiogenic shock. Bratisl. Lek. Listy.

[9] Li S., Xiao F.-Y., Shan P.-R., Su L., Chen D.-L., Ding J.-Y., Wang Z.-Q. (2015). Overexpression of microRNA-133a inhibits ischemia-reperfusion-induced cardiomyocyte apoptosis by targeting DAPK2. J. Hum. Genet..

[10] Yu B.-T., Yu N., Wang Y., Zhang H., Wan K., Sun X., Zhang C.-S. (2019). Role of miR-133a in regulating TGF-β1 signaling pathway in myocardial fibrosis after acute myocardial infarction in rats. Eur. Rev. Med. Pharmacol. Sci..

[11] Horie T., Ono K., Nishi H., Iwanaga Y., Nagao K., Kinoshita M., Kuwabara Y., Takanabe R., Hasegawa K., Kita T. (2009). MicroRNA-133 regulates the expression of GLUT4 by targeting KLF15 and is involved in metabolic control in cardiac myocytes. Biochem. Biophys. Res. Commun..

[12] Chen Y., Zhao Y., Chen W., Xie L., Zhao Z.-A., Yang J., Chen Y., Lei W., Shen Z. (2017). MicroRNA-133 overexpression promotes the therapeutic efficacy of mesenchymal stem cells on acute myocardial infarction. Stem Cell Res. Ther..

[13] Song T., Yao Y., Wang T., Huang H., Xia H. (2017). Tanshinone IIA ameliorates apoptosis of myocardiocytes by up-regulation of miR-133 and suppression of Caspase-9. Eur. J. Pharmacol..

[14] He B., Xiao J., Ren A.-J., Zhang Y.-F., Zhang H., Chen M., Xie B., Gao X.-G., Wang Y.-W. (2011). Role of miR-1 and miR-133a in myocardial ischemic postconditioning. J. Biomed. Sci..

[15] Bostjancic E., Zidar N., Stajer D., Glavac D. (2012). MicroRNAs, miR-1, miR-133a/b and miR-208 in infarcted and remote myocardium of human myocardial infarction with the focus on the ventricular fibrilation and/or tachycardia. FEBS J..

[16] Xu C., Hu Y., Hou L., Ju J., Li X., Du N., Guan X., Liu Z., Zhang T., Qin W. (2014). β-Blocker carvedilol protects cardiomyocytes against oxidative stress-induced apoptosis by up-regulating miR-133 expression. J. Mol. Cell. Cardiol..

[17] Muniyandi P., Palaninathan V., Mizuki T., Maekawa T., Hanajiri T., Mohamed M.S. (2020). Poly(lactic-*co*-glycolic acid)/polyethylenimine nanocarriers for direct genetic reprogramming of microRNA targeting cardiac fibroblasts. ACS Appl. Nano Mater..

[18] Ma D., Liu H., Zhao P., Ye L., Zou H., Zhao X., Dai H., Kong X., Liu P. (2020). Programing assembling/releasing multifunctional miRNA nanomedicine to treat prostate cancer. ACS Appl. Mater. Interfaces.

[19] Cai X.X., Zhu Q.X., Zeng Y., Zeng Q., Chen X.L., Zhan Y.H. (2019). Manganese oxide nanoparticles as MRI contrast agents in tumor multimodal imaging and therapy. Int. J. Nanomed..

[20] Ferreira M.P.A., Talman V., Torrieri G., Liu D., Marques G., Moslova K., Liu Z., Pinto J.F., Hirvonen J., Ruskoaho H. (2018). Dual-drug delivery using dextran-functionalized nanoparticles targeting cardiac fibroblasts for cellular reprogramming. Adv. Funct. Mater..

[21] Zhang S., Wang J., Pan J. (2016). Baicalin-loaded PEGylated lipid nanoparticles: Characterization, pharmacokinetics, and protective effects on acute myocardial ischemia in rats. Drug Deliv..

[22] Duro-Castano A., Gallon E., Decker C., Vicent M.J. (2017). Modulating angiogenesis with integrin-targeted nanomedicines. Adv. Drug Deliv. Rev..

[23] Wang Y., Wang X., Zhang Y., Yang S., Wang J., Zhang X., Zhang Q. (2009). RGD-modified polymeric micelles as potential carriers for targeted delivery to integrin-overexpressing tumor vasculature and tumor cells. J. Drug Target..

[24] Zhao L., Yang C., Dou J., Xi Y., Lou H., Zhai G. (2015). Development of RGD-functionalized PEG-PLA micelles for delivery of curcumin. J. Biomed. Nanotechnol..

[25] Hao R., Sun B., Yang L., Ma C., Li S. (2020). RVG29-modified microRNA-loaded nanoparticles improve ischemic brain injury by nasal delivery. Drug Deliv..

[26] Li D., Zhou J., Yang B., Yu Y. (2019). MicroRNA-340-5p inhibits hypoxia/reoxygenation-induced apoptosis and oxidative stress in cardiomyocytes by regulating the Act1/NF-κB pathway. J. Cell. Biochem..

[27] Geng X.-Y., Xiao N., Han Y., Li Y.-J. (2019). Platelet microparticles: A tool to predict infarction area in rats. J. Investig. Surg..

[28] Jin H., Li D.Y., Chernogubova E., Sun C., Busch A., Eken S.M., Saliba-Gustafsson P., Winter H., Winski G., Raaz U. (2018). Local delivery of miR-21 stabilizes fibrous caps in vulnerable atherosclerotic lesions. Mol. Ther..

[29] Cook R.L., Householder K.T., Chung E.P., Prakapenka A.V., DiPerna D.M., Sirianni R.W. (2015). A critical evaluation of drug delivery from ligand modified nanoparticles: Confounding small molecule distribution and efficacy in the central nervous system. J. Control. Release.

[30] Menown I.B.A., Mackenzie G., Adgey A.A.J. (2000). Optimizing the initial 12-lead electrocardiographic diagnosis of acute myocardial infarction. Eur. Heart J..

[31] Koentges C., Bode C., Bugger H. (2016). SIRT3 in cardiac physiology and disease. Front. Cardiovasc. Med..

[32] Mitchelson K.R., Qin W.-Y. (2015). Roles of the canonical myomiRs mi R-1,-133 and-206 in cell development and disease. World J. Biol. Chem..

[33] Xu M., Xue R.-Q., Lu Y., Yong S.-Y., Wu Q., Cui Y.-L., Zuo X.-T., Yu X.-J., Zhao M., Zang W.-J. (2018). Choline ameliorates cardiac hypertrophy by regulating metabolic remodelling and UPRmt through SIRT3-AMPK pathway. Cardiovasc. Res..

[34] Zhang L., Wu Y., Li Y., Xu C., Li X., Zhu D., Zhang Y., Xing S., Wang H., Zhang Z. (2012). Tanshinone IIA improves miR-133 expression through MAPK ERK1/2 pathway in hypoxic cardiac myocytes. Cell. Physiol. Biochem..

[35] Wang L., Li X., Zhou Y., Shi H., Xu C., He H., Wang S., Xiong X., Zhang Y., Du Z. (2014). Downregulation of miR-133 via MAPK/ERK signaling pathway involved in nicotine-induced cardiomyocyte apoptosis. Naunyn. Schmiedebergs. Arch. Pharmacol..

[36] Yu C., Xue J., Zhu W., Jiao Y., Zhang S., Cao J. (2015). Warburg meets non-coding RNAs: The emerging role of ncRNA in regulating the glucose metabolism of cancer cells. Tumor Biol..

[37] Klishadi M.S., Zarei F., Hejazian S.H., Moradi A. (2015). Losartan protects the heart against ischemia reperfusion injury: Sirtuin3 involvement. J. Pharm. Pharm. Sci..

[38] Bheri S., Davis M.E. (2019). Nanoparticle-hydrogel system for post-myocardial infarction delivery of microRNA. ACS Nano.

[39] Curaj A., Simsekyilmaz S., Staudt M., Liehn E. (2015). Minimal invasive surgical procedure of inducing myocardial infarction in mice. J. Vis. Exp..

[40] Wang C., Jing Q. (2018). Non-coding RNAs as biomarkers for acute myocardial infarction. Acta Pharmacol. Sin..

[41] Quagliariello V., Coppola C., Mita D.G., Piscopo G., Iaffaioli R.V., Botti G., Maurea N. (2019). Low doses of Bisphenol A have pro-inflammatory and pro-oxidant effects, stimulate lipid peroxidation and increase the cardiotoxicity of Doxorubicin in cardiomyoblasts. Environ. Toxicol. Pharmacol..

[42] Jang E.H., Shim M.K., Kim G.L., Kim S., Kang H., Kim J.-H. (2020). Hypoxia-responsive folic acid conjugated glycol chitosan nanoparticle for enhanced tumor targeting treatment. Int. J. Pharm..

