# Supplementary material for: RGD-PEG-PLA Delivers MiR-133 to Infarct Lesions of Acute Myocardial Infarction Model Rats for Cardiac Protection
Source: Pharmaceutics. 2020 Jun 21;12(6):575. doi: 10.3390/pharmaceutics12060575 (PMC7356814; doi:10.3390/pharmaceutics12060575)
Supplement: Supplementary file 1 [file pharmaceutics-12-00575-s001.docx]

Supplementary Materials: RGD-PEG-PLA Delivers MiR-133 to Infarct Lesions of Acute Myocardial Infarction Model Rats for Cardiac Protection

Bixi Sun, Shuwen Liu, Rubin Hao, Xinyue Dong, Lanbo Fu, and Bing Han

This supplementary material contains:

1) The table format of the size and zeta potential characterizations of different nanoparticles (Table S1)

2) The table format of typical changes of electrocardiogram in different groups (Table S2)

**Table S1.** Size and zeta potential characterizations of different nanoparticles.

| **Grouping** | **Size (nm)** | **Zeta Potential (mV)** |
| --- | --- | --- |
| PEG-PLA/miR-133 | 94.8 ± 14.1 | −11.9 ± 1.5 |
| RGD-PEG-PLA/miR-133 | 137.9 ± 9.7 | −4.9 ± 1.1 |

**Table S2.** Typical changes of electrocardiogram in different groups.

| **Grouping** | **Heart Rate (bmp)** | **ST-Segment Elevation** | **Pathological Q Waves** |
| --- | --- | --- | --- |
| Sham | 330 | - | - |
| Model | 414 | + + + | + + + |
| Positive drug | 388 | + + | + + |
| MiR-133 | 345 | + | + |
| PEG-PLA/miR-133 | 395 | + + | + + |
| RGD-PEG-PLA/miR-133 | 362 | + | + |
